# Suicidal and non-suicidal self-injurious behaviour in patients with bipolar disorder and comorbid attention deficit hyperactivity disorder after initiation of central stimulant treatment: a mirror-image study based on the LiSIE retrospective cohort

**DOI:** 10.1177/2045125320947502

**Published:** 2020-08-06

**Authors:** Louise Öhlund, Michael Ott, Robert Lundqvist, Mikael Sandlund, Ellinor Salander Renberg, Ursula Werneke

**Affiliations:** Department of Clinical Sciences, Division of Psychiatry, Sunderby Research Unit, Umeå University, Umeå, 901 87, Sweden; Department of Public Health and Clinical Medicine, Division of Medicine, Umeå University, Umeå, Sweden; Department of Public Health and Clinical Medicine, Sunderby Research Unit, Umeå University, Luleå, Sweden; Department of Clinical Sciences, Division of Psychiatry, Umeå University, Umeå, Sweden; Department of Clinical Sciences, Division of Psychiatry, Umeå University, Umeå, Sweden; Department of Clinical Sciences, Division of Psychiatry, Sunderby Research Unit, Umeå University, Luleå, Sweden

**Keywords:** attention deficit disorder with hyperactivity, bipolar disorder, central nervous system stimulants, non-suicidal self injury, self-injurious behaviour, suicide, suicide attempted

## Abstract

**Background:**

Currently, our understanding regarding treatment of adult attention deficit hyperactivity disorder (ADHD) co-occurring with bipolar disorder (BD) remains limited. The aim of this study was to evaluate the impact of central stimulant (CS) treatment on suicidal and non-suicidal self-injurious behaviour in patients with a pre-existing diagnosis of BD or schizoaffective disorder (SZD). Specifically, we tested the hypothesis that CS treatment significantly decreased the number of suicide attempts and non-suicidal self-injury events.

**Methods:**

A mirror-image study in patients with a dual diagnosis of BD or SZD and ADHD, comparing suicide attempts and non-suicidal self-injury events within 6 months and 2 years before and after CS initiation. This study was part of a retrospective cohort study (LiSIE) into effects and side-effects of lithium for maintenance treatment of BD as compared with other mood stabilisers.

**Results:**

Of 1564 eligible patients, 206 patients met the inclusion criteria. Within the 6 months after CS initiation, suicide attempts and non-suicidal self-injury events decreased significantly, both in terms of numbers of patients having such events (*p* = 0.013) and numbers of events experienced (*p* = 0.004). These effects were preserved 2 years after CS initiation.

**Conclusions:**

CS treatment may reduce the risk of suicide attempts and non-suicidal self-injury events in patients with a dual diagnosis of BD or SZD and ADHD. Based on our findings, clinicians should not withhold CS treatment from patients with concomitant ADHD for fear of deterioration of the underlying BD. However, to minimise the risk of manic episodes concomitant mood stabiliser treatment and close monitoring remains warranted.

## Introduction

Adult attention deficit hyperactivity disorder (ADHD) has emerged as a clinically significant comorbidity in patients with bipolar disorder (BD). Both disorders can present with similar symptoms, such as hyperactivity, impulsivity and disinhibition. This overlap of symptoms can make it difficult to distinguish both disorders.^1^ Thus, it comes to no surprise that reported prevalence figures for the co-occurrence of both disorders (dual diagnosis) vary greatly. For individuals with BD, comorbid ADHD prevalence estimates range from 4% to 48%.^2,3^ For individuals with ADHD, comorbid BD prevalence estimates range from 5% to 47%.^4–7^ Co-occurrence of BD and ADHD is associated with unfavourable outcomes in terms of unemployment, lower socioeconomic status, unstable relationships, substance abuse, increased risk of hypomania and suicide attempts (SA).^8^ ADHD in itself seems to increase the risk of SA.^9–11^ The risk may increase even further in the presence of comorbid BD,^9,10,12,13^ since BD *per se* already carries a high risk of suicidal behaviour. Up to 35% of individuals with type I BD (BD-I) and 41% of individuals with type II BD (BD-II) have a history of SA.^2^

At present, our understanding is limited about how both conditions should be treated when occurring together. Neither do we know in how many, currently adult, patients with BD a diagnosis of ADHD has been missed due to overlapping symptoms and ADHD being a relatively novel diagnosis. This question is of high clinical relevance, since patients with BD and comorbid ADHD may benefit from central stimulants (CS) such as methylphenidate or amphetamine preparations. Yet, relatively little is known about the safety aspects of using CS in patients with a dual diagnosis of BD and ADHD. When treating this vulnerable patient group, relapse and suicide prevention will always be on the forefront of the clinician’s mind. Concerning prevention of relapse risk, current evidence suggests that CS treatment may be safe. A large Swedish register study found that methylphenidate could be used safely in adults with BD, as long as mood-stabilisers were given simultaneously to prevent manic episodes.^14^ A Swedish register study in adolescents or young adults treated with methylphenidate found no evidence that initiation of methylphenidate treatment increased the risk of psychotic events. This held true for both patients with and without a prior history of psychosis. However, this study did not explore BD as a comorbidity.^15^ Concerning prevention of suicidal and non-suicidal self-injurious behaviour, CS do not seem to increase such risk in patients with only ADHD.^16,17^ Rather, CS may reduce the risk.^9,17,18^ Yet, in patients who suffer from both BD and ADHD, the impact of CS on the risk of suicidal and non-suicidal self-injurious behaviour remains poorly understood. Neither is it known how concurrent mood stabiliser treatment modifies the impact of CS on suicidal behaviour. This question is particularly relevant in patients who receive antipsychotics as mood stabilisers, because these may antagonise the pro-dopaminergic effects of CS.^19^

### Aim of this study

The aim of this study was to evaluate the impact of CS treatment on SA and non-suicidal self-injury (NSSI) events (henceforth referred to as SA/NSSI events) in patients with a pre-existing diagnosis of BD or schizoaffective disorder (SZD). Specifically, we tested the hypothesis that CS treatment significantly decreased the number of SA/NSSI events.

## Methods

**Figure 1.**
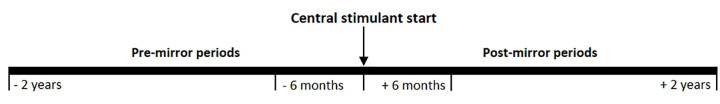
Study design.

We designed a retrospective cohort study (Lithium Effects and Side Effects/LiSIE) to (a) explore effects and side-effects of lithium for the maintenance treatment of BD as compared with other mood stabilisers, and (b) identify the best long-term treatment options for patients with BD and related conditions under consideration of potential comorbidities.^20^ The Regional Ethics Review Board at Umeå University, Sweden, has approved this study (DNR 2010-227-31M, DNR 2011-228-32M, DNR 2014-10-32M, 2018-76-32M). This particular study concerned the impact of CS treatment in patients who had a current diagnosis of BD or SZD. We used a mirror-image design to compare the frequency of SA/NSSI events within 6 months and 2 years before CS initiation (pre-mirror periods) and within 6 months and 2 years after CS initiation (post-mirror periods) (Figure 1). For included patients, we retrospectively examined routine clinical data from the electronic medical records and electronic prescriptions linked to the medical records from 1997 up to 31 December 2017. Electronic case records have comprehensively been in existence in the studied catchment area (Region Norrbotten) since 1997. No individual pharmacy dispensing records were available to us.

### Participants

#### LiSIE cohort

For the LiSIE cohort, we invited all individuals in the two northernmost Swedish regions (Norrbotten and Västerbotten) who were at least 18 years old and who had either received a diagnosis of BD (ICD F31), SZD (ICD F25) or had used lithium as mood stabiliser between 1997 and 2011. The participants were informed about the nature of the study in writing and provided verbal informed consent. The consent was documented in our research files, dated and signed by the research worker who obtained the consent. In accordance with the ethics approval granted, for deceased patients, no consent was obtained. Consent procedures concluded by the end of 2012. The cohort was locked at this point, and no new patients were included into the study thereafter.

#### Patient selection and inclusion criteria

From this cohort, we identified all patients in the Swedish region of Norrbotten of (a) at least 18 years of age, who (b) had received a diagnosis of BD or SZD on at least two occasions, at least 6 months apart any time between 1997 and 2013, and (c) who had received a diagnosis of ADHD (F90) any time between 1997 and 2017. We included patients who (a) had obtained a comorbid diagnosis of ADHD after having received a diagnosis of BD or SZD, (b) had maintained their diagnosis of BD or SZD at CS initiation, and (c) had started CS treatment at any time until 31 December 2015. This time point was chosen to allow 2-year coverage of the post-mirror period after CS initiation.

#### Exclusion criteria

For the whole LiSIE study, we excluded patients in whom, after manual validation from the medical records, a diagnosis of schizophrenia was more likely than BD or SZD. For this particular study, we also excluded patients, who (a) had received CS before the age of 18 years, (b) had received CS before the diagnosis of BD or SZD, and (c) for whom the affective diagnosis or its timing could not be confirmed before CS initiation.

### Variable definitions

#### Outcome

Our outcome was SA/NSSI events, within 6 months and 2 years before and after initiation of CS. These periods, we refer to as pre-mirror and post-mirror periods (Figure 1). Information regarding deliberate self-harm, regardless of intention, was retrieved from psychiatric records. This means that both SA and NSSI events were monitored.^21^ Events were abstracted from the medical records when patients came directly to psychiatric services in connection with the event or through consultation requests or emergency referrals. The following events were included: (a) SA with a clear stated suicide intent; (b) NSSI events; (c) events where the intention was unclear and could be either a SA or a NSSI; and (d) deliberate, self-inflicted self-poisoning events, where the intention was unclear and could be either a SA or NSSI. Thus, self-poisoning was considered a NSSI when the intention was stated as an action of self-harm without suicidal intent. Also, occurrence of suicide within the 2 years after CS initiation was recorded and confirmed by the Swedish Cause of Death register.^22^ Intoxications were excluded, when explicitly relating to non-suicidal harmful substance use or addiction.

### Exposures

#### Diagnosis

In the medical records, clinicians had used diagnoses either according to the Diagnostic and Statistical Manual of Mental Disorders (DSM) or the International Classification of Diseases (ICD) in their various editions. We obtained the diagnoses including working diagnoses of BD/SZD and ADHD within 2 years before CS initiation. We validated these diagnoses from the clinical accounts manually to reach approximations of what, according to DSM-5,^23^ the most likely diagnoses would have looked like. Affective diagnoses included BD-I, SZD, BD-II and other BD. Other BD included unspecified BD or BD type IV. BD type IV was used when hypomanic episodes had been induced by antidepressants. We divided these affective diagnoses into two categories: BD-I/SZD and BD-II/other BD. Patients with rapid cycling or episodes with mixed features were allocated to either group depending on the underlying diagnosis. A presence of a manic episode automatically allocated a patient to the BD-I/SZD group.

ADHD diagnoses included ADHD, attention deficit disorder (ADD) or unspecified hyperactivity disorder. Where explicitly stated, we recorded the point of time of the ADHD diagnosis. We recorded whether ADHD rating scales and/or neuropsychological assessments had been used in the context of CS initiation. Method of diagnosis was divided in three categories, (a) diagnosis obtained after neuropsychological assessment, (b) clinical diagnosis based on psychiatric assessment complemented by psychometric rating scales, and (c) clinical diagnosis based on psychiatric assessments only.

#### Central stimulants

The index event in our study was the first prescription of CS. As CS, we included methylphenidate extended and immediate release, lisdexamphetamine, dexamphetamine and amphetamine. We recorded type and dose at the point of, and 2 years after, CS initiation. When a CS was discontinued before the end of the 2-year period, we used type and dose at the point of discontinuation. When CS was discontinued and subsequently reinstated, a mean dose was calculated accordingly. Based on extended release CS formulations available in Sweden, we divided doses into four categories, (a) low (starting), (b) intermediate, (c) high, and (d) exceeding the highest recommended dose in adults according to the Swedish national formulary.^24^ Where patients also received immediate release formulations, we added these to the extended dose recommendations. For United Kingdom (UK) reference, we checked the Swedish dose recommendations against the British National Formulary (BNF) (Table 1).^25^ To account for discrepancies between the highest recommended doses in Sweden and the UK, we amalgamated these four categories into two groups, (a) low for low or intermediate dose, and (b) high for high dose or a dose exceeding the highest recommended dose.

**Table 1.** Central stimulant extended release formulations and dosing for adults.^24,25^

Active ingredient	Methylphenidate				Lisdexamphetamine
Central stimulant brand used	Concerta**®**	Equasym**®**	Medikinet**®**	Ritalin**®**	Elvanse**®**
*Dose categories based on Swedish National Formulary*					
**Low (starting), mg**	18	10–20	10–20	10–20	30
**Intermediate, mg**	27–36	30–40	30–60	30–60	50
**High, mg**	54	50–60	70–80	70–80	70
**Highest recommended dose, mg**	54	60	80	80	70
*For comparison: dose recommendations derived from the NICE BNF*					
**Highest recommended dose, mg**	108	100	100	Not available	70

BNF, *British National Formulary*; NICE, National Institute for Health and Care Excellence.

#### CS discontinuation

We also checked whether CS had been discontinued during the post-mirror period. Some patients had discontinued and then reinstated CS on at least one occasion. CS discontinuation was used as a proxy to distinguish continuous, ‘stable’ CS treatment from episodic, ‘unstable’ treatment. The total duration of CS treatment during the 2-year follow-up was calculated for each patient.

#### Mood stabilisers

We recorded mood stabilisers used in the pre- and post-mirror periods. Here, we considered lithium, anticonvulsants, including sodium valproate, lamotrigine, carbamazepine and topiramate. We also considered second-generation antipsychotics (SGA), including olanzapine, quetiapine, aripiprazole and risperidone. We then categorized mood stabilisers in three groups; (a) lithium, (b) anticonvulsants and (c) SGA.

#### Other ADHD medications

We also recorded use of other ADHD medications, including atomoxetine, or CS like treatments, such as modafinil, prior to CS initiation. As only a negligible number of patients (*n* = 11) had received other ADHD medications, we did not explore such further.

#### Hospital admissions

We systematically abstracted the number of psychiatric hospital admissions in the pre- and post-mirror periods. Main reasons for admissions were also recorded. Hospital admissions were used as a proxy for severe relapses in the pre-and post-mirror periods.

#### Alcohol and substance use

We explored alcohol and substance misuse within the 2-year pre- and post-mirror periods. We recorded alcohol and/or substance misuse, when (a) there was an alcohol or substance use disorder diagnosed according to DSM or ICD in their various editions, or (b) there was an explicit reference in the medical records.

### Data collection and analysis

The clinical data were abstracted into a database by one of the investigators (LÖ). Data abstraction was fact-based as recorded in the medical records, using the previously mentioned variable definitions to minimise interpretation. If SA or NSSI was not stated in the medical records, an event was not counted. Unclear events were discussed in the research group. All SA and NSSI events were amalgamated into one group of SA/NSSI events to minimise misclassification according to intent. Data were then anonymised before analysis. In a first step, we analysed data descriptively, establishing the frequency of all variables in our database. Means and standard deviations were calculated for continuous data and frequencies and percentages for categorical data. We then compared SA/NSSI events in the pre- and post-mirror periods. Here we compared (a) number of patients who had experienced such events and (b) number of events per patient. As the data were not normally distributed, we used non-parametric tests for pairwise (before/after) comparisons. For these pairwise comparisons, we used McNemar’s exact test for the number of patients with SA/NSSI events and Wilcoxon signed rank test for the numbers of events.

In addition, we explored potential confounding factors that might have been associated with SA/NSSI events within the 2-year pre- and post-mirror periods taken together (whole 4-year review period) with a generalised linear mixed model (GLMM). We considered the following factors: period (respective pre-and post-mirror period), age, sex, type of underlying affective disorder, CS dose (high/low), mood stabilisers (never, used only in pre-mirror period, only in the post-mirror period or in both periods), alcohol or substance misuse (never, only in pre-mirror period, only in the post-mirror period or in both periods), and CS discontinued in post-mirror episode. With this set-up it was possible to analyse (a) our outcome, that is, counts of events, and (b) repeated observations for each individual, that is, the number of events before and after the first administration of CS. The GLMM was set-up using a negative binomial distribution and a log link, and individual patients were modelled as a random effect. This is explained in further detail in the SPSS syntax given in Appendix 1. Throughout, statistical significance level was set to *p* < 0.05. For the statistical analysis, we used the IBM SPSS version 26 (IBM, Armonk, NY, USA).

#### Control for bias

A total of 75% percent of all approached patients consented to inclusion into the LiSIE study. In accordance with the ethical approval granted, we controlled for selection bias in the whole LiSIE study, comparing age, sex, maximum recorded serum lithium and creatinine levels as key parameters, available in anonymised form. There were no significant differences between participating and non-participating patients.

## Results

### Description of sample

**Figure 2.**
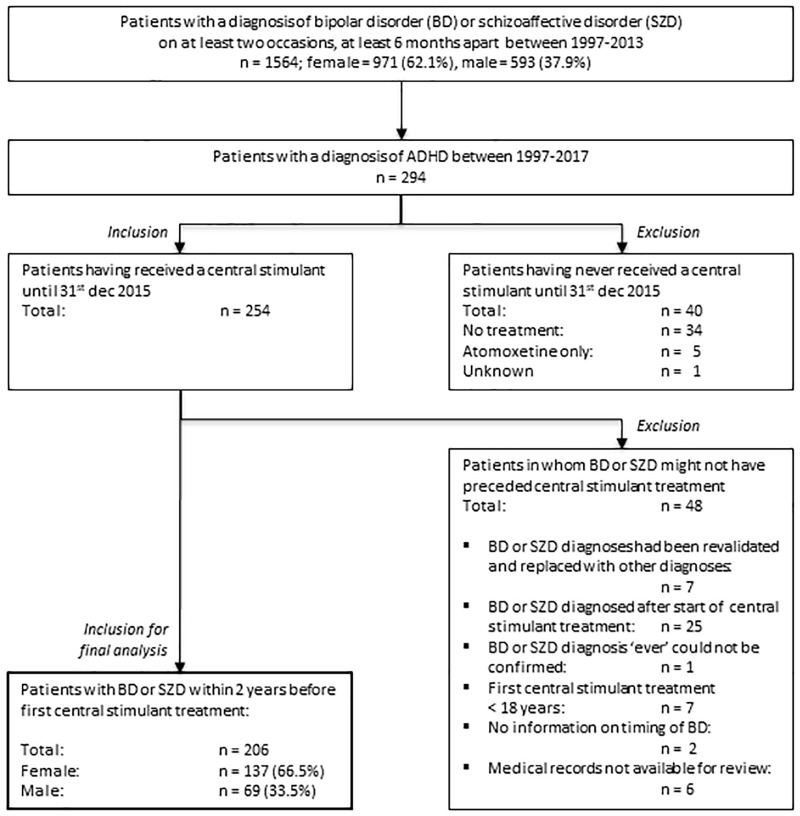
Identification of study sample.

Of 1564 patients in the LiSIE cohort diagnosed with BD or SZD, 294 (19%) had also received a diagnosis of ADHD at some point. 206 (13%) patients met the inclusion criteria (Figure 2). The earliest date of CS initiation was 15 May 2002, the latest date of CS initiation was 21 October 2015.

In our sample, 66% of patients were female; 91% had a diagnosis of BD-II/other BD (Table 2). For six patients, data for the either pre-or post-mirror periods was incomplete.

**Table 2.** Baseline characteristics of patients with BD or SZD and ADHD at the first CS treatment episode.

	Total sample
*n*	206
Gender, *n* (%)	
Female	137 (66.5)
Male	69 (33.5)
Age at CS start	
Mean (SD)	34.6 (10.8)
Median (min–max)	33.0 (18–61)
Type of disorder within the 2-year pre-mirror period (underlying affective disorder), *n* (%)	
BD-I/SZD	19 (9.2)
BD-I	14 (6.8)
SZD	5 (2.4)
BD-II/other BD	187 (90.8)
BD-II	135 (65.5)
Other BD	52 (25.2)
Type of ADHD diagnosis at CS start, *n* (%)	
ADHD	155 (75.2)
ADD	32 (15.5)
Unspecified hyperactivity disorder	6 (2.9)
ADHD diagnosis documented later	13 (6.3)
Method of diagnosing ADHD, *n* (%)	
Neuropsychological assessment	38 (18.4)
Clinical + rating scales	136 (66.0)
Clinical only	27 (13.1)
Unknown	5 (2.4)
Use of ADHD rating scale prior CS start, *n* (%)^a^	167 (81.1)
Type of CS treatment at initiation, *n* (%)	
Methylphenidate extended release	201 (97.6)
Methylphenidate immediate release	4 (1.9)
Amphetamine/lisdexamphetamine preparation	1 (0.5)
Previous treatment with atomoxetine ever, *n* (%)	10 (4.9)
Previous treatment with modafinil ever, *n* (%)	1 (0.5)
Type of CS treatment at last follow-up within the 2-year post-mirror period	
Methylphenidate extended release	189 (91.7)
Methylphenidate immediate release	3 (1.5)
Methylphenidate extended + immediate release	5 (2.4)
Amphetamine/lisdexamphetamine preparation	9 (4.4)
CS dose at last follow-up within the 2-year post-mirror period	
High	91 (44.2)
Low	115 (55.8)
CS discontinued any time within the 2-year post-mirror period, *n* (%)	102 (49.5)
CS treatment at the end of the 2-year post-mirror period, *n* (%)	128 (62.1)
Total time on CS treatment during the 2-year post-mirror period, months	
Mean (SD)	17.2 (8.5)
Median (min–max)	22.9 (0.10–24.0)
Mood stabiliser use within the 2-year pre- and post-mirror periods, *n* (%)	
Lithium in both periods	40 (19.4)
Lithium in pre-mirror period only	22 (10.7)
Lithium in post-mirror period only	14 (6.8)
No lithium treatment	130 (63.1)
SGA in both periods	52 (25.2)
SGA in pre-mirror period only	38 (18.4)
SGA in post-mirror period only	40 (19.4)
No SGA treatment	76 (36.9)
Anticonvulsant in both periods	82 (39.8)
Anticonvulsant in pre-mirror period only	38 (18.4)
Anticonvulsant in post-period only	20 (9.7)
No anticonvulsant treatment	66 (32.0)
Alcohol and/or substance misuse within the 2-year pre- and post-mirror periods, *n* (%)	49 (23.8)
In both periods	28 (13.6)
In pre-mirror period only	15 (7.3)
In post-mirror period only	6 (2.9)
No alcohol and/or substance misuse	157 (76.2)

ADD, attention deficit disorder; ADHD, adult attention deficit hyperactivity disorder; ASRS, Adult ADHD Self-Report Scale; BD, bipolar disorder; BD-I, bipolar disorder type I; BD-II, bipolar disorder type II; CS, central stimulant; n, number; other BD, unspecified or other specified bipolar disorder; SD, standard deviation; SGA, second generation antipsychotic; SZD, schizoaffective disorder; WURS, Wender Utah Rating Scale.

a Mainly WURS and ASRS.

All proportions rounded to 1 decimal point, due to rounding % may not always add up perfectly to 100%.

### CS treatment

The mean age at the beginning of CS treatment was 35 (SD 11) years. All but one patient had received methylphenidate as first ADHD medicine. At the end of the last follow up within the 2-year period, 115 (56%) of patients were treated with low dose of CS and 91 (44%) were treated with high dose of CS. All but nine patients received methylphenidate. A total of 49.5% of patients had discontinued CS treatment at some point during the 2-year post-mirror period; 24 (12%) of patients subsequently reinstated CS and were still treated with CS at their last follow up. In total, 62% of patients were treated with CS at their last follow up. The total mean duration of CS treatment during the 2-year follow up was 17 (SD 8) months with a median of 23 months (Table 2).

### Mood stabiliser treatment

Overall, mood-stabiliser use did not change between the pre- and post-mirror periods (*p* = 0.780). Regarding individual substances/substance classes, there was no significant difference in the 2-year pre-and post-mirror periods for lithium (*p* = 0.243) and for SGA (*p* = 0.910). Anticonvulsant use decreased significantly in the post-mirror period (*p* = 0.025).

### SA/NSSI events before and after CS initiation

For the 6-month mirror period, complete data was available for 204 patients. For the 2-year mirror period, complete data was available for 200 patients. During the 6-month pre- and post-mirror periods, pair-wise comparison showed that 14 patients had SA/NSSI events before and 4 after CS initiation (*p* = 0.013) (Table 3). The number of SA/NSSI events decreased from 20 to 5. The intra-individual change of number of events was significantly different between periods (*p* = 0.004).

**Table 3.** Patients with SA and NSSI events before and after CS initiation.

Within 6 months (*n* = 204)				
		**After** ^a^		
		Yes	No	Total
**Before**	Yes	2	12	14
	No	2	188	190
	Total	4	200	204
Within 2 years (*n* = 200)				
		After^b^		
		Yes	No	Total
**Before**	Yes	8	27	35
	No	13	152	165
	Total	21	179	200

CS, central stimulant; NSSI, non-suicidal self-injury; SA, suicide attempts.

a *p* = 0.013.

b *p* = 0.038.

During the 2-year pre-and post-mirror periods, pair-wise comparison showed that 35 patients had SA/NSSI events before and 21 after CS initiation (*p* = 0.038) (Table 3). The number of SA/NSSI events dropped from 52 to 31. The intra-individual change of number of events was significantly different between episodes (*p* = 0.028).

After adjusting for multiple potential confounders in the whole sample, the mean number of SA/NSSI events were still significantly fewer in the post-mirror period (OR 0.63; 95% CI 0.40–0.98, *p* = 0.041) (Table 4).

**Table 4.** Factors associated with SA and NSSI events within 2 years before and after CS initiation.

Dependent variable: number of SA/NSSI events	Coefficient	SE	OR	95% CI		*p*
				Lower	Upper	
**Post-mirror period** ^a^	**−0.465**	**0.227**	**0.63**	**0.40**	**0.98**	**0.041**
Pre-mirror period^b^ (baseline)						
Gender						
Male	−0.687	0.461	0.50	0.20	1.25	0.137
Female (baseline)						
**Age at CS start, mean**	**−0.097**	**0.025**	**0.91**	**0.86**	**0.95**	**<0.001**
Type of underlying affective disorder						
**BD-I/SZD**	**−1.528**	**0.755**	**0.22**	**0.05**	**0.96**	**0.044**
BD-II/other BD (baseline)						
Alcohol and/or substance misuse within the pre- and post-mirror periods						
**In both pre- and post-mirror periods**	**1.259**	**0.547**	**3.52**	**1.20**	**10.31**	**0.022**
**In pre-mirror period only**	**1.325**	**0.462**	**3.76**	**1.52**	**9.33**	**0.004**
In post-mirror period only	0.155	1.337	1.17	0.08	16.18	0.908
No alcohol and/or substance misuse (baseline)						
CS dose at last follow-up within the post-mirror period						
High	0.411	0.393	1.51	0.70	3.27	0.296
Low (baseline)						
Mood stabiliser use within the pre-and post-mirror periods						
Lithium in both pre- and post-mirror period	0.758	0.472	2.14	0.84	5.40	0.109
Lithium in pre-mirror period only	1.047	0.614	2.85	0.85	9.52	0.089
Lithium in post-mirror period only	0.266	0.926	1.31	0.21	8.07	0.774
No lithium treatment (baseline)						
**SGA in both pre- and post-mirror period**	**1.798**	**0.492**	**6.04**	**2.29**	**15.88**	**<0.001**
**SGA in pre-mirror period only**	**1.163**	**0.518**	**3.20**	**1.16**	**8.86**	**0.025**
**SGA in post-mirror period only**	**1.475**	**0.585**	**4.37**	**1.38**	**13.81**	**0.012**
No SGA treatment (baseline)						
Anticonvulsant in both pre- and post-mirror period	0.835	0.492	2.30	0.88	6.06	0.091
Anticonvulsant in pre-mirror period only	0.069	0.561	1.07	0.36	3.23	0.902
Anticonvulsant in post-period only	−0.239	0.701	0.79	0.20	3.12	0.733
No anticonvulsant treatment (baseline)						
CS discontinued at any time within the post-mirror period						
Yes	0.071	0.401	1.07	0.49	2.36	0.859
No (baseline)						

BD-I, bipolar disorder type I; BD-II, bipolar disorder type II; CS, central stimulant; OR, odds ratio; other BD, unspecified or other specified bipolar disorder; *p, p*-value; SA/NSSI, suicide attempts and non-suicidal self-injury; SE, standard error; SGA, second generation antipsychotics; SZD, schizoaffective disorder.

a The 2-year period after start of CS treatment.

b The 2-year period before start of CS treatment.

In addition, this model showed other factors with significant effect on the number of SA/NSSI events. Such events decreased with age. There were fewer events in the BD-I/SZD group. There were more events in patients with alcohol or substance misuse and in patients receiving SGA (Table 4).

### Hospital admissions

During the 6-month pre- and post-mirror periods, pair-wise comparison showed that 31 patients were admitted for psychiatric in-patient care before and 19 after CS initiation (*p* = 0.029). The number of admissions decreased from 50 to 39. However, there was no significant intra-individual change in number of admissions between periods (*p* = 0.071). There were four admissions due to mania/hypomania before and three admissions after CS initiation.

During the 2-year pre-and post-mirror periods, pair-wise comparison showed that 54 patients were admitted for psychiatric in-patient care before and 46 after CS initiation (*p* = 0.291). The number of admissions decreased from 135 to 123. However, there was no significant intra-individual change in number of admissions between periods (*p* = 0.669). There were eight hospital admissions due to mania/hypomania before and nine after CS initiation.

## Discussion

### Main findings

The main finding of our study was that SA/NSSI events decreased significantly within 6 months after CS initiation. This suggests that in patients with BD and ADHD, CS initiation could swiftly alleviate extreme suffering leading to suicidal behaviour. This effect was even maintained after 2 years of CS initiation, irrespective of whether CS had been discontinued or not. The reasons for this observed effect remain unclear. On the one hand, the decreased number of SA/NSSI events can reflect a direct CS effect; on the other, it is possible that patients were in a more stable phase of their BD so that clinicians felt sufficiently safe to start CS to treat comorbid ADHD. Prospective studies are required to address this question. The decrease in number of SA/NSSI events did not come at the expense of more admissions. Any such increase in admissions might have pointed to more manic episodes or an increased need for suicide prevention. Both would have diluted the beneficial effect of CS initiation. To our knowledge, this is the first study ever evaluating effects of CS on suicidal behaviour in patients with BD and ADHD. Our study adds further support to findings that CS can safely be used in patients with a dual diagnosis of BD and ADHD. However, as 91% of included patients had a diagnosis of BD-II/other BD, our results are applicable mainly to this group and cannot automatically be extrapolated to patients with BD-I/SZD.^26^ As previously pointed out by others, mood stabilisers should be used concomitantly to prevent manic episodes.^14^ An incidental finding from our study was that SGA were positively associated with SA/NSSI events, already before, but even more so after CS initiation. The reasons for this finding remain unclear. Patients who receive antipsychotic medication may be more severely ill and hence also more vulnerable to adverse effects of CS. At the same time, CS by virtue of dopamine agonistic effects may revert at least partly the dopamine-2 receptor (D2) blockade of antipsychotics.^19^ The question of whether CS and antipsychotics should be used in conjunction remains controversial and requires further study.^27,28^

### Comparison with other studies

#### Prevalence of comorbid bipolar disorder and ADHD

In our study, 19% of patients diagnosed with BD/SZD, had also received a diagnosis of ADHD at some point. However, not all patients had both diagnoses at the same time; 13% received an ADHD diagnosis after having received a diagnosis of BD/SZD. This suggests that in our cohort, the ‘true’ prevalence of a dual diagnosis of BD/SZD and ADHD, treated and untreated, lay somewhere in between 13% and 19%. This prevalence lies well in the range of that reported in other studies (Table 5).^2,3,29–42^ Half of these other studies report more male participants, the other half reports more female participants. In our study, in keeping with the gender distribution in the entire LiSIE cohort, there were more women than men. In most of the other reviewed studies, BD and ADHD, BD-I exceeds BD-II. But there are also studies that report greater prevalence of BD-II.^2,33,38^ In one study, BD-I was more frequent in patients with non-rapid cycling BD and BD-II more frequent in patients with rapid cycling BD.^31^ In our study, the BD-I/SZD to BD-II/other BD ratio was 0.1. At present, we lack an explanation for these differences in prevalence of gender distribution and BD types in patients with a dual diagnosis of BD and ADHD. Possibly, women are more likely to be diagnosed with BD first, before ADHD is considered. This may then delay the ADHD diagnosis into adulthood.^4^ Rapid cycling implies a more erratic clinical course, complicating the distinction from BD. Most prevalence studies are cross-sectional. Such do not pick up diagnostic changes over time. BD-II tends to be more common in patients with ADHD diagnosed in adulthood. This may reflect a more heterogenous clinical course with greater impairment from depressive symptoms, more emotional dysregulation, subthreshold symptoms and loss of functionality.^4,43^

**Table 5.** Prevalence of co-occurrence of ADHD in adults with BD reported since 2001.

	Country	Type of study	Time frame	Sample, *n* and BD type	Prevalence of co-occurrence	BD-I/ BD-II ratio	Male/ female ratio
Karanti *et al*.^2^	Sweden	Cross-sectional	-	8463	3.7%	0.7	-
Pinna *et al*.^8^	Italy	Prospective	15 years	703	24.6%	1.4	1.9
Gomes *et al*.,^29^	Brazil	Cross-sectional	-	64	17.2%	BD-I only	-
Chen *et al*.^30^	Sweden	Cross-sectional	-	1,665,938	14.3%^a^	-	0.9
Aedo *et al*.^31^	Chile	Cross-sectional	-	Total: 235 NRC: 191 RC: 44	Total: 9.8% NRC: 8.3% RC: 23.7%	Total^b^: NRC: 2.3 RC: 0.72	Total: 0.6 NRC: 0.7 RC: 0.3
Harmanci *et al*.^3^	Turkey	Cross-sectional	-	100	48.0%	4.3	-
Torres *et al*.^32^	Spain	Cross-sectional	-	163	10.4%	1.8	2.4
Perroud *et al*.^33^	Switzerland	Cross-sectional	-	138	19.5%	0.1	0.8
Karaahmet *et al*.^34^	Turkey	Cross-sectional	-	90	Adulthood: 23.3% Childhood: 14.4%	-	Adulthood: 2.5 Childhood: 1.6
Perugi *et al*.^35^	Italy	Cross-sectional	-	96	19.8%	2.8	2.7
Bernardi *et al*.^36^	Italy	Cross-sectional	-	100	Lifetime: 18.0% Current: 10.0%	-	2.6
McIntyre *et al*.^37^	Canada	Cross-sectional	-	176	Lifetime: 17.6%	No significant difference	0.6
Rydén *et al*.^38^	Sweden	Cross-sectional	-	159	Adulthood: 16.3% Childhood: 11.9%	Adulthood: 0.7^c^ Childhood: 0.7^c^	Adulthood: 0.6 Childhood: 0.6
Sentissi *et al*.^39^	France	Cross-sectional		73	Adult: 30.1%	3.4	-
Tamam *et al*.^40^	Turkey	Cross-sectional	-	44	15.9%	BD-I only	0.4
Kessler *et al*.^41^	USA	Cross-sectional		3199	21.2%		
Nierenberg *et al*.^42^	USA	Cross-sectional	-	919	9.5%^c^	4.8	1.8

ADHD: attention deficit hyperactivity disorder; BD: bipolar disorder; BD-I, bipolar disorder type I; BD-II, bipolar disorder typ II; n, number; NOS, BD not otherwise specified; NRC, non-rapid cycling; RC, rapid cycling.

a Age = 18–64 years.

b Two patients with NOS excluded.

c BD-II + BD NOS.

### Suicidal behaviour in patients with a dual diagnosis of BD and ADHD

In patients with BD, comorbid ADHD has been identified as a risk factor for suicidal behaviour. This increased risk has been found in adults overall,^3,8,44^ as well as in young adults and adolescents.^12^ However, one study did not find a significant association between ADHD and SA in adolescents with BD.^45^ In patients with ADHD, comorbid BD has also been identified as a risk factor for suicidal behaviour in adults overall,^13^ and in young adults and adolescents.^9^ Since most studies point towards an increased risk of suicidal behaviour in patients with a dual diagnosis of BD and ADHD, the question arises of whether CS treatment can reduce this risk in these patients. This question has not been explored in the literature. Current research has explored the impact of CS on the risk of suicidal behaviour in patients with only ADHD or as an adjunctive treatment in patients with bipolar depression. In our study, SA/NSSI events were more common among patients with BD-II/other BD and ADHD than among patients with BD-I/SZD and ADHD. This is consistent with findings that suggest a higher suicide risk in BD-II than in BD-I. The reasons for this are not quite clear. A higher occurrence of agitated depression and anxiety may play a role,^26,46^ factors that could not be explored further in our study.

### Risk of suicidal behaviour with CS in patients with only ADHD

According to the national formularies in the UK and Sweden, methylphenidate is contraindicated in patients with suicidal tendencies.^24,25^ The Swedish national formulary suggests that suicidal thoughts may appear in about 0.1% to 1% treated patients. SA, including completed suicides, are much rarer with <0.01%.^24^ For lisdexamphetamine, suicidal tendencies are not listed as a contraindication; neither are suicidal thoughts or acts listed in the adverse effect profile.^24^ Given the paramount importance of CS in the treatment of ADHD, it is important to understand the implications for suicidal thoughts and acts. Available studies point to CS being risk-neutral or associated with a decreased risk. A large Swedish register study of adult patients found no association between CS treatment (methylphenidate, dexamphetamine or amphetamine) and concomitant suicidal behaviour. In within-patient comparison, the results pointed towards a protective effect.^17^ More evidence is available for children and adolescents. A nationwide study of children and young adults with ADHD from Taiwan found a significant reduction in suicide risk in patients having been prescribed methylphenidate for more than 90 days. The risk was even more reduced in patients having been prescribed methylphenidate for more than 180 days. In patients having been prescribed methylphenidate for 90 days or less, there was no change in suicide risk.^16^ A study of children and adolescents in Hong Kong showed that the risk of SA peaked before methylphenidate treatment. It remained high immediately after treatment start and then declined with continuous treatment.^18^ A study, based on insurance claims data from Taiwan, found methylphenidate risk-neutral in regard to SA of adolescents and young adults. For males, however, the risk of SA decreased with higher methylphenidate doses.^9^ Another study following 97 boys with ADHD into young adulthood made a similar observation; those treated with higher methylphenidate doses had significantly fewer SA during childhood.^47^ In a study based on the Italian ADHD register, suicidal ideation was not observed as an adverse event in 1350 children and adolescents treated with methylphenidate over a 5-year period.^48^ Finally, a US insurance claims study of 223,303 patients between 5 and 17 years of age, who had used CS as a first-line ADHD treatment, confirmed that suicidal events (attempt or completed suicide) were rare, 26.3 per 100,000 person years during current CS use and 32.6 per 100,000 person years during former CS use.^49^

### Adjunctive CS treatment in patients with BD

In patients with BD, depressive symptoms have been associated with an increased risk of suicidal behaviour.^50–52^ This makes patients with BD-II disorder particularly vulnerable.^2,44^ CS have been suggested as an adjunctive treatment for BD,^52,53^ which may modify the risk of suicide. In the context of BD, most trials have been conducted with modafinil or armodafinil. There are only few studies and case series that explore adjunctive use of methylphenidate and lisdexamphetamine.^52–57^ The small numbers of participants in these studies mean that suicide risk as a potential adverse effect cannot be addressed. The use of CS in bipolar depression remains poorly understood. Current studies focus on the risk of acute relapses rather than risk of suicidal behaviour.^58^

### Reduction of impulsivity as a means to reduce suicidal or non-suicidal self-injurious behaviour

The mechanism by which CS could reduce suicidal or NSSI behaviour also remains largely unexplored. Suicidal behaviour has been linked to impulsive aggression,^59^ impaired executive function and inhibition,^60^ and altered decision-making.^61^ These factors have also been observed in patients with BD. Impaired executive function, particularly impaired decision making and impulsivity,^62–66^ has been linked to an increased risk of suicidal and NSSI behaviour. Similar observations have been made in patients with ADHD. One study of 539 patients who had attempted suicide showed that SA were not associated with ADHD *per se* but with impulsive aggression, particularly when there were ADHD symptoms.^67^ In patients with ADHD, treatment with CS such as methylphenidate reduces impulsivity and aggressive behavior.^68,69^ Methylphenidate increases dopamine and norepinephrine activity through reuptake inhibition of both neurotransmitters. Amphetamines such as lisdexamphetamine increase dopamine and norepinephrine activity through reuptake inhibition and release of both neurotransmitters.^69,70^ CS increase catecholamine availability in striatal and cortical regions. This may improve executive function, emotional responsivity, regulation of reward processes and risky decision making.^69^

### Simultaneous use of CS and antipsychotics

Whereas CS enhance activity at the dopamine 2 (D2) receptor, antipsychotics reduce D2 activity. Thus, they should cancel out each other’s action, thereby causing a ‘dopamine dilemma’ in patients who use CS and antipsychotics simultaneously.^19^ However, little is known about the effects and adverse effects of such combinations. The available evidence is heterogenous and inconclusive. MRI studies in adolescents with ADHD suggest that methylphenidate may normalise structural fronto-striatal brain changes but that concurrent antipsychotic treatment may counteract these structural improvements.^69,71^ A systematic review of concurrent use of CS and antipsychotics in children and adolescents did demonstrate neither benefits nor an excess in adverse events with combination therapy.^28^ Another study suggested that administration of low dose amphetamines might improve cognitive function in patients with schizophrenia who received appropriate antipsychotic treatment.^72^ A recent Swedish register study explored the risk of psychosis after methylphenidate initiation in individuals aged between 12 and 30 years with a previous history of psychosis. There was no significant change in risk of psychotic events when comparing the 12-week period before and after starting methylphenidate. However, this study did not assess concomitant use of methylphenidate and antipsychotics. Hence the study could not determine whether the risk of psychotic events was moderated by antipsychotic treatment.^15^

## Strengths

Our study was not based on register data but real-life detailed clinical data at symptoms and treatments levels. This allowed us to study SA/NSSI events as an outcome of high clinical relevance that was not easily obtainable from register data. We could validate all exposure variables from the case records, including diagnoses and CS exposure. Access to data from the medical records permitted distinction between the various types of BD and exclusion of other diagnoses including schizophrenia. As clinical response and propensity to adverse effects may differ between the various types of BD, it is important to distinguish between BD-I and BD-II. We could also determine date of CS start and stop and CS doses with a much higher accuracy than possible with register data.

The mirror-image design, used to assess SA/NSSI events before and after CS initiation, allowed individuals to act as their own controls. In such way, other factors known to be associated with worse prognosis in patients with BD and ADHD could be adjusted within the design.^12,40,42^ This minimised confounding at individual level.^73,74^ We also adjusted for potential confounders in our GLMM.

A further strength was the high rate of consent for participation into the study. Age and sex distribution of consenting and non-consenting patients was similar. Therefore, the likelihood of selection bias is low.

## Limitations

Our study had an observational design in a naturalistic setting, exploring the effects of CS initiation retrospectively. We did not compare patients with a dual diagnosis of BD/SZD and ADHD who started CS with a control group of patients with BD/SZD and ADHD who did not use CS. The non-randomised design limited our ability to draw affirmative conclusions about the benefits and risks of CS treatment in patients with BD. Yet, randomised controlled trials, particularly when sample sizes are small and follow up times are short, may not be able to pick up SA/NSSI events as an outcome in the same way as our retrospective study could.

One limitation relates to the quantification of self-inflicted events. All SA/NSSI events were counted as recorded in the medical notes, irrespective of whether ICD-codes for intentional self-harm (X60 – X84)^75^ were recorded or not. Reliance on ICD-encodings for intentional self-harm could have led to an underestimation of self-harm events. We could only include SA/NSSI events when the patient had been in contact with health services. Thus, we may have underestimated the number of events. However, more serious attempts could be expected to have led to contact with clinical services and hence been captured by our study. We slightly deviated from the general definition of NSSI. This refers to deliberate, self-inflicted destruction of the body, such as cutting, burning or scratching oneself,^21,76,77^ but not to self-poisoning events. We included self-poisoning in order to capture overdoses with stated self-harm intent, which would otherwise have been missed.

The quality of our data depended on the quality of the information in the medical notes. As all information was collated from medical notes, we could not apply Structured Clinical Interview Tool for DSM Disorders (SCID) or similar tools to validate the diagnoses. The starting point of our study was BD, that is, we only included patients who received CS as adults after they had received a diagnosis of BD. We excluded patients who first had received CS and then a diagnosis of BD. As we did not have access to the child and adolescent psychiatric records, we could not check for ADHD diagnosis during childhood. Yet, the childhood history was mostly summarised in the adult records. There is also a possibility that some patients with BD never received a diagnosis of ADHD, because their symptoms were seen as part of their BD. Here, the question arises whether ADHD was mistaken as BD. Besides, ADHD may have been mistaken for other psychiatric comorbidities.^78^

However, access to data at symptom level, reduced the potential for misclassification beyond what is possible in observational studies based on register data. We employed several measures to minimise misclassification and observer bias; (a) the data abstraction was conducted along predefined variables; (b) only events were counted that had been explicitly recorded in the medical notes; and (c) all unclear events were discussed in the research group. This way, we followed procedures used by other published studies in the field.^79–81^ However, even when following standardised procedures, in many cases we were not able to distinguish between SA and NSSI. In the records, we found that the attending clinicians had difficulties to make this distinction and did not always agree. Although patients sometimes changed their own views whether self-injury had happened with suicidal intent or not. Therefore, we could reliably establish that a SA/NSSI event had happened. However, we could not always establish whether there had been suicidal intent. Therefore, we amalgamated SA and NSSI. Difficulties to distinguish between SA and NSSI are a commonly encountered problem in clinical studies.^82^

Our observed prevalence lay in the range reported by other studies. In any event, we did not examine the impact of the diagnosis of ADHD *per se* but the impact of CS use as a proxy for ADHD.

The prescription of CS was clearly documented in the medical records or the electronic prescriptions linked to the medical record. We cannot exclude that some patients still may occasionally have received a traditional prescription issued on paper. Neither could we establish with certainty whether patients used CS beyond of what was prescribed and, if so, to which extent. Both could have led to an underestimation of CS use. But, as CS prescriptions were also documented in the medical records, we judge the risk for underestimation as low.

However, the scope for underestimation of comorbidities such as anxiety and borderline personality syndrome was greater, because this was not consistently documented. Therefore, we did not judge this data to be sufficiently reliable for adjustment in our model. For some patients, data was missing in either pre- or post-mirror period. However, as data was complete for >97% of patients, this is unlikely to have distorted the results. It was impossible to know whether patients really adhered to their prescribed medication. Finally, alcohol and substance misuse could only be detected if the patient sought care and may have been underestimated.

## Conclusion

CS treatment may reduce the risk of SA/NSSI events in patients with a dual diagnosis of BD and ADHD. This finding is of substantial clinical significance but requires replication in further prospective studies. The decrease in number of SA/NSSI events did not come at the expense of more frequent admissions. Based on our findings, clinicians should not withhold CS treatment from patients with concomitant ADHD for fear of deterioration of the underlying BD. However, to minimise the risk of manic episodes and to err on the side of caution, concomitant mood stabiliser treatment and close monitoring remains warranted.^14^ The benefit and risks of concomitant use of CS and antipsychotics requires further clarification.

## Supplemental Material

Appendix_1 – Supplemental material for Suicidal and non-suicidal self-injurious behaviour in patients with bipolar disorder and comorbid attention deficit hyperactivity disorder after initiation of central stimulant treatment: a mirror-image study based on the LiSIE retrospective cohort Click here for additional data file. Supplemental material, Appendix_1 for Suicidal and non-suicidal self-injurious behaviour in patients with bipolar disorder and comorbid attention deficit hyperactivity disorder after initiation of central stimulant treatment: a mirror-image study based on the LiSIE retrospective cohort by Louise Öhlund, Michael Ott, Robert Lundqvist, Mikael Sandlund, Ellinor Salander Renberg and Ursula Werneke in Therapeutic Advances in Psychopharmacology
