# Supplementary material for: Suicidal and non-suicidal self-injurious behaviour in patients with bipolar disorder and comorbid attention deficit hyperactivity disorder after initiation of central stimulant treatment: a mirror-image study based on the LiSIE retrospective cohort
Source: Ther Adv Psychopharmacol. 2020 Aug 6;10:2045125320947502. doi: 10.1177/2045125320947502 (PMC7418477; doi:10.1177/2045125320947502)
Supplement: Appendix_1 – Supplemental material for Suicidal and non-suicidal self-injurious behaviour in patients with bipolar disorder and comorbid attention deficit hyperactivity disorder after initiation of central stimulant treatment: a mirror-image study based on the LiSIE retrospective cohort [file Appendix_1.pdf]

Appendix 1: Syntax

Code book

Name in SPSS	Nr of levels	Label	Role
count_selfharm		Number of suicide attempts/non-suicidal self-injury events	Outcome
patient		Patient nr	
period	2	Period, either the two-year mirror period before (pre-mirror) or the two-year mirror period after (post-mirror) CS initiation	Factor
gender	2		Factor
age		Age at beginning of CS treatment	Covariate
alcsubst	2	Alcohol and/or substance misuse within the pre- and post-mirror periods, (a) diagnosed according to DSM or ICD, or (b) an explicit reference in the medical records.	Factor
dose	2	(a) CS dose at the end of the post-mirror period, (b) the dose at CS discontinuation or, (c) the mean CS dose for those who discontinued and subsequently reinstated CS. Coded as Low or High	Factor
disc	2	CS discontinued at any time within the post-mirror period. Yes/ No	Factor
LI	4	Lithium at any time in pre- and/or post-mirror periods, not pre/not post, pre/not post, not pre/post, pre/post	Factor
SGA	4	SGA at any time in pre- and/or post-mirror periods, not pre/not post, pre/not post, not pre/post, pre/post	Factor
AC	4	Anticonvulsant at any time in pre- and/or post-mirror periods, not pre/not post, pre/not post, not pre/post, pre/post	Factor

bp	2	Type of underlying affective disorder within the pre-mirror period. BD-I/SZD or BD-II/other BD	Factor

Syntax for GLMM

GENLINMIXED

/DATA_STRUCTURE SUBJECTS=patient REPEATED_MEASURES=period COVARIANCE_TYPE=IDENTITY

/FIELDS TARGET=count_selfharm TRIALS=NONE OFFSET=NONE

/TARGET_OPTIONS DISTRIBUTION=NEGATIVE_BINOMIAL LINK=LOG

/FIXED EFFECTS= period gender age alcsubst dose disc LI SGA AC bp USE_INTERCEPT=TRUE

/RANDOM USE_INTERCEPT=TRUE SUBJECTS=patient COVARIANCE_TYPE=IDENTITY SOLUTION=FALSE

/BUILD_OPTIONS TARGET_CATEGORY_ORDER=ASCENDING
INPUTS_CATEGORY_ORDER=DESCENDING

MAX_ITERATIONS=100 CONFIDENCE_LEVEL=95 DF_METHOD=RESIDUAL COVB=ROBUST
PCONVERGE=0.000001(ABSOLUTE)

SCORING=0 SINGULAR=0.000000000001
